# Evaluation of bladder filling effects on the dose distribution during radiotherapy for cervical cancer based on daily CT images

**DOI:** 10.1002/acm2.14097

**Published:** 2023-07-12

**Authors:** Fan Zhang, Mi Zhou, Gang Wang, Xutong Li, Lu Yue, Lihua Deng, Kun Chi, Kai Chen, Zhenyu Qi, Xiaowu Deng, Yinglin Peng, Yimei Liu

**Affiliations:** ^1^ Department of Radiation Oncology State Key Laboratory of Oncology in South China Guangdong Key Laboratory of Nasopharyngeal Carcinoma Diagnosis and Therapy Sun Yat‐sen University Cancer Center Guangzhou China; ^2^ Department of Radiation Oncology Qingdao Municipal Hospital Qingdao China

**Keywords:** bladder filling, cervical cancer, daily CT, dose distribution, radiotherapy

## Abstract

**Purpose:**

This study aimed to assess the effects of bladder filling during cervical cancer radiotherapy on target volume and organs at risk (OARs) dose based on daily computed tomography (daily‐CT) images and provide bladder‐volume‐based dose prediction models.

**Methods:**

Nineteen patients (475 daily‐CTs) comprised the study group, and five patients comprised the validation set (25 daily‐CTs). Target volumes and OARs were delineated on daily‐CT images and the treatment plan was recalculated accordingly. The deviation from the planning bladder volume (DVB), the correlation between DVB and clinical (CTV)/planning (PTV) target volume in terms of prescribed dose coverage, and the relationship of small bowel volume and bladder dose with the ratio of bladder volume (RVB) were analyzed.

**Results:**

In all cases, the prescribed dose coverage in the CTV was >95% when DVB was <200 cm^3^, whereas that in the PTV was >95% when RVB was <160%. The ratio of bladder V_45 Gy_ to the planning bladder V_45 Gy_ (RBV_45_) exhibited a negative linear relationship with RVB (RBV_45_ = −0.18*RVB + 120.8; *R* ^2^ = 0.80). Moreover, the ratio of small bowel volume to planning small bowel volume (RVS) exhibited a negative linear relationship with RVB (RVS = −1.06*RVB +217.59; *R* ^2^ = 0.41). The validation set results showed that the linear model predicted well the effects of bladder volume changes on target volume coverage and bladder dose.

**Conclusions:**

This study assessed dosimetry and volume effects of bladder filling on target and OARs based on daily‐CT images. We established a quantitative relationship between these parameters, providing dose prediction models for cervical cancer radiotherapy.

## 1 INTRODUCTION

Intensity‐modulated radiotherapy (IMRT) is a standard treatment for patients with cervical cancer and is also frequently combined with brachytherapy, chemotherapy, and thermotherapy to provide better patient outcomes.^1^, ^2^ IMRT reduces the probability of complications in the gastrointestinal tract, bone marrow, and genitourinary systems compared with conventional three‐dimensional conformal radiotherapy due to the high conformality of the target volume and prescribed dose and the rapid dose falloff outside the target volume.^3^, ^4^, ^5^ However, patients with cervical cancer experience changes in bladder filling during IMRT; this results in relative changes to the relative position of the target volumes and organs at risk (OARs), leading to uncertainty in the actual irradiation doses to both the target volume and OARs and significantly affecting the accuracy of radiotherapy for cervical cancer.^6^

Although the patients undergo bladder preparation, all of them exhibit poor bladder volume reproducibility, resulting in wide variations in bladder volume, from 7% to 450% relative to baseline.^6^ Jhingran et al. found that despite instructing patients to hold their urine during treatment, twice‐weekly cone‐beam CT (CBCT) results showed a median difference of 247 cm^3^ between the minimum and maximum bladder volumes.^7^ A recent study conducted by Li et al. using four‐dimensional magnetic resonance imaging (MRI) also found significant changes in bladder volume and uterine position between scans.^8^ Another study conducted by Eminowicz recorded the distance (mm) from the PTV edge to the CTV to examine whether CTV was fully covered by the PTV. When the bladder volume difference is > 130 cm^3^ from planning, CTV was not covered by PTV; when the bladder volume is > 300 cm^3^ higher at the time of planning, the actual bladder volume at treatment may not be reproducible.^9^ In addition, the degree of bladder filling at the time of daily treatment has a substantial effect on the small bowel dose. Approximately 20% of unintentional dose delivered to the small bowel is attributed to variations in bladder filling; moreover, the physiological peristalsis of the small bowel also increases the uncertainty of the dose received.^10^ Jadon et al. systematically reviewed several previous studies on intrapelvic organ motility in cervical cancer patients treated with radiotherapy and observed that patient bladder volume affects the accuracy of target localization.^11^ However, none of these studies conducted an investigation into the relationship between bladder volume and its associated variability and the dosimetry of target and OAR during the treatment course.

Currently, most clinical studies are based on CBCT images to guide radiotherapy for cervical cancer; thus, most studies evaluated the organ motion and set‐up errors in radiotherapy based on the CBCT findings.^9^, ^12^, ^13^ However, due to insufficient FOV scanning and poor image quality of CBCT images, the range of dose calculation may be insufficient, and it is difficult to accurately define and delineate the target volume and OARs. Currently, the uRT‐linac506c (United Imaging Healthcare, Shanghai, China) linear accelerator combines a spiral computed tomography, which can perform a fan‐beam CT scan before each treatment, resulting in a diagnostic grade, high‐quality CT image.^14^ Target volumes and OARs contouring and dose calculation were completed in the daily online CT image, thereby providing a useful tool for the assessment of changes in the location of target volumes and OARs and dosimetric changes in cervical cancer radiotherapy. This device can be used for routine image‐guided radiotherapy and adaptive radiotherapy.

In this study, we aimed to examine the volume changes in the bladder and small bowel during cervical cancer radiotherapy by acquiring and analyzing the daily online CT images obtained using the uRT‐linac506c linear accelerator. We also aimed to further analyze the relationship between bladder volume and small bowel volume, as well as its relationship with dosimetric changes in the target volume, to elucidate the effects of bladder volume changes in the dosimetry of the target volume and surrounding OARs, thereby providing dose predictions related to bladder volume for cervical cancer radiotherapy.

## 2 METHODS

### 2.1 Patients

Twenty‐four patients with cervical cancer (median age: 55 years, range: 36−68 years) who had been treated at our cancer center between September 2021 and April 2022 were selected. Disease staging was performed according to the International Federation of Gynecology and Obstetrics guidelines; of the total patients, 5, 12, and 7 were classified as having stages II, III, and IV, respectively. The 24 patients were randomly divided into two groups (study group: 19 and validation group: 5). The study was approved by the Institutional Review Board of the Sun Yat‐sen University Cancer Center (No. B2017‐008‐01), and all patients provided written informed consent.

### 2.2 Imaging acquisition

#### 2.2.1 CT simulation

Patients were placed in the supine position with a vacuum pad for postural immobilization. All patients were asked to empty their bladder and rectum 1 hour before the CT simulation scanning (Philips Brilliance Big Bore), and the water intake was for the 500−1000 mL volume in one time after emptying. A handheld ultrasound instrument (Verathon Bladder scan 9400) was used to measure urine volume of patient, and if it is less than 200 cm^3^, the patient should continue preparing until it is greater than 200 cm^3^. CT simulation scanning was performed when the patient's bladder was prepared. All patients were scanned using a Philips 64‐channel spiral large‐aperture CT simulator based on the following parameters: 140‐kV voltage, 300‐mAs current, 3‐mm scan slice thickness, 3‐mm reconstruction slice thickness, and 0.688‐cm pitch, and a scan range from the inferior border of the first lumbar vertebra to 5 cm below the ischial tuberosity. Moreover, all patients received a dose of about 13.54mGy per scan. The acquired CT images were transferred to a radiotherapy planning system (Monaco, V5.01, Elekta AB, Stockholm, Sweden) for delineation.

#### 2.2.2 Daily‐CT

The uRT linac has a diagnostic‐quality helical CT system compactly fixed behind the gantry of a C‐arm linac, and the patient is sent through the scanner by moving the couch longitudinally. Compared with kV‐CBCT that is commonly used for IGRT, the helical kV fan‐beam CT is nearly free from degrading of photon scattering, providing a slice‐to‐slice comparison of patient's anatomy with planning CT. Prior to each treatment, the patient was again instructed to hold urine and empty rectum to reproduce the state of the CT simulation scan. The patients underwent laser positioning and were scanned before each treatment. The scan range was same with that of planning CT (pCT) using the following scanning conditions: voltage, 120 kV; current, 70 mAs; scan slice thickness, 3 mm; reconstruction slice thickness, 3 mm; and pitch, 0.688 cm. All scans were performed in low‐dose mode, with the dose per scan being approximately about 4.74mGy. The obtained CT images were rigidly aligned to bony anatomy of the planning CT images to minimize effect from set‐up errors. A total of 500 online CT scans from the 24 patients were included in this study, with 475 scans in the study group and 25 scans in the validation group. Nineteen patients in the study group had one pCT scan and 25 treatment CT scans per patient, whereas five patients in the validation group had one pCT scan and 5 weekly treatment CT scans per patient.

### 2.3 Target volume and OAR delineation

The target volumes and OARs were manually delineated by two MD physicians on the patient's pCT images using the Monaco treatment planning system (V5.01, Elekta AB) based on clinical requirements. The target volume included metastatic lymph nodes (GTVnd) and clinical target volume (CTV). CTV included the primary tumor, cervix, uterus, proximal vagina identified by physical examination and radiology (according to the region of tumor invasion), parametrial tissue, and lymph node CTV (included external iliac, internal iliac, obturator foramen, presacral, and common iliac), paraaortic and inguinal lymphatic drainage area (accorded to the patient's specific conditions). Taking into account set‐up error and organ motion, the corresponding planning target volumes (PGTVnd and PTV) were generated by extending 0.5 cm outward in each direction. The delineated OARs mainly include the bladder, rectum, small intestine and spinal cord. After delineation, the treatment planning CT images and online daily‐CT images of each patient were imported into the uRT Treatment Planning System (Version 3.2) for analysis.

### 2.4 Treatment planning

#### 2.4.1 Treatment planning

Planning design was performed using dual‐arc volumetric modulated arc therapy or fixed‐field IMRT. The X‐ray energy was 6 MV, and the prescribed PTV dose was 45–50 Gy. The dosimetric requirements were as follows: approximately 100% of the prescribed dose covering 95% of the PTV, the exposure dose of OARs should be limited as far as possible while ensuring the coverage of the target area.

#### 2.4.2 Daily planning

Based on the rigid alignment results of the planning CT and daily‐CT images, the treatment plan was recalculated, but not optimized, based on the daily‐CT images to determine the dose, and the individual plan parameters were confirmed to be consistent with the treatment plan parameters by QA (quality assurance) dose calculation which calculates the original plan on the rigidly registered online CT.

### 2.5 Data recording and processing

**FIGURE 1:**
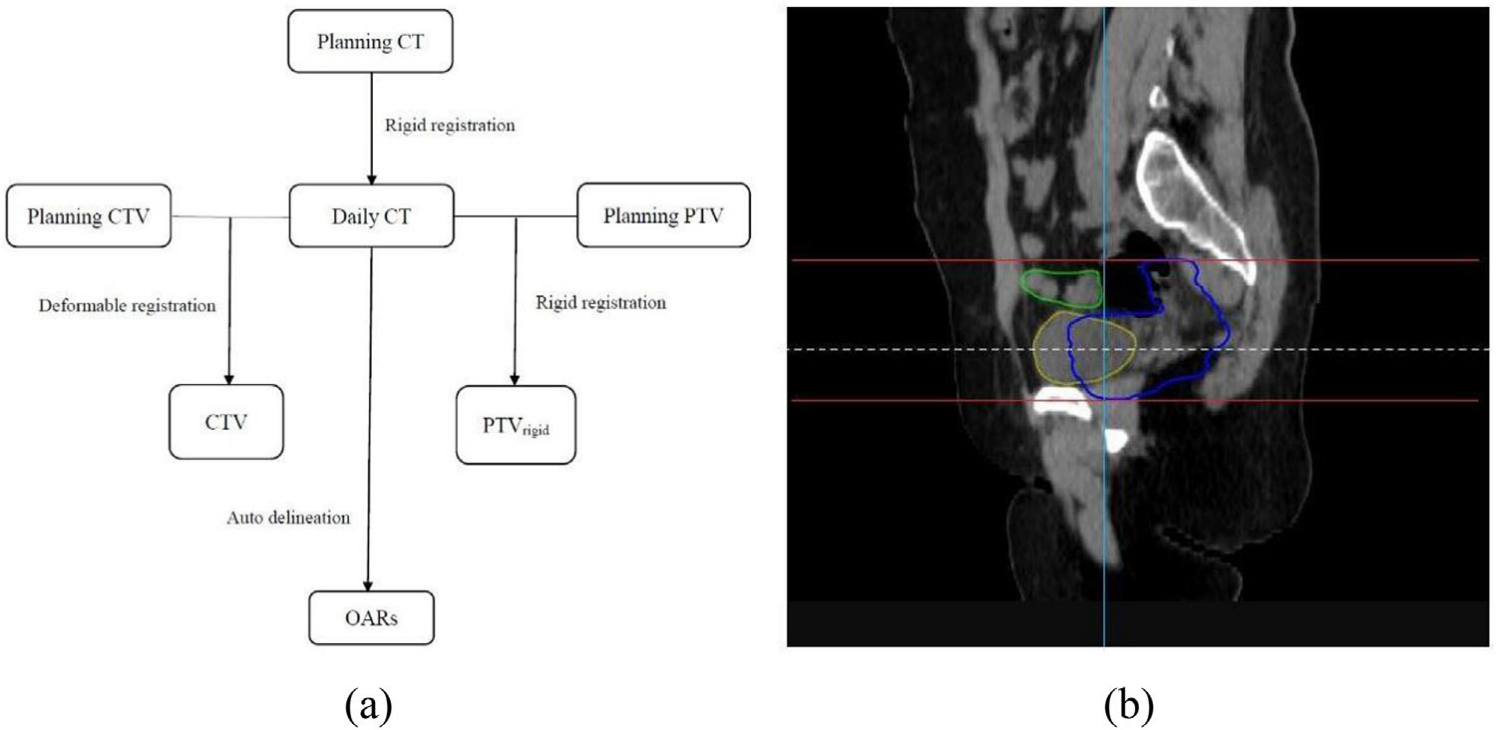
(a) Flowchart of the target volume and OAR delineation. (b) A sagittal plane of the bladder, target, and small bowel. The red lines indicate the top and bottom boundaries of the PTV. CT = computed tomography; CTV = clinical target volume; OAR = organ at risk; PTV = planning target volume.

To study the patient's organ motion per treatment and the target volume dose coverage, a retrospective analysis of the daily‐CT was performed with the following scheme (see also Figure 1a). (1) Image registration: The planning CT images were registered along with the daily‐CT images based on the bony matching results at the time of treatment. (2) Target volume and OAR delineation: The uRT planning system uses three algorithms (deformation registration, rigid registration, and automatic delineation). For the target volume, the CTV was deformed to the daily‐CT images through the deformation registration process and modified and reviewed by the same two physicians; The planning PTV was directly copied to the daily‐CT image through rigid registration to form PTV_rigid_. For OARs, automatic delineation was performed to generate outlines of the bladder, small bowel, and the left and right femoral heads, which were subsequently modified and reviewed by the same two radiologists. (3) To reduce the influence of the target volume and OARs (e.g. small bowel) at other sites far away from bladder on the results, a specific range of body volume was selected. The lower boundary of interested volume is the lower boundary of the target volume of the planning CT and the upper boundary of interested volume is 1.5 cm above the upper margin of the bladder of the planning CT (Figure 1b). (4) The dose, volume, and centroid of the target volume and the OARs were calculated using the planning system. The data were processed in the same manner for both study and validation groups. All data and graphs were derived from the statistical analysis using Origin (Version 8.0, OriginLab, USA).

### 2.6 Data analysis

#### 2.6.1 Changes in bladder volume during radiotherapy

The maximum bladder volume at treatment for each patient was defined as VB_max_, the minimum volume as VB_min_, the mean volume as VB_mean_, and the planning bladder volume as VB_plan_. The deviation from the planning bladder volume (DVB) and the ratio of bladder volume to planning bladder volume (RVB) were used to analyze the change in bladder volume during radiotherapy (VB_treat_) in comparison to the planning bladder volume (VB_plan_) for each patient. The DVB and RVB of the study and validation groups were calculated using the following formulas:

$$\begin{array}{c} \mathrm{DVB}=VB_{\mathrm{treat}}-VB_{\mathrm{plan}}\\ \mathrm{RVB}=\left({\mathrm{VB}}_{\mathrm{treat}}/VB_{\mathrm{plan}}\right)\times 100\% \end{array}$$

The RVBs of the patients were ranked, and the mean RVB was counted in an interval of 10%; the width of the total statistical interval was 10−210%. To better fit the correlation linear curve, a small number of cases in which VB_treat_ were considerably larger than VB_plan_ (RVB ≥ 210%) were not considered. The cases we omitted were not reproducible, taking a small proportion of the dataset (5.9%, 28/475).

#### 2.6.2 Correlation between bladder filling and target volume dose coverage

The impact of absolute deviation in bladder volume on target volume dose coverage was analyzed. For each patient, the dose (not normalized) of CTV, PTV_rigid_ was classified into two groups by whether or not V100% > 95%. Then, taken the target prescription coverage in planned CT as reference, the coverage of CTV, PTV_rigid_ of patients at each fraction was normalized according to planned prescription coverage respectively. The target volume coverage was fitted linearly to the mean RVB correspondingly to investigate the correlation between them.

#### 2.6.3 Correlation between bladder filling and OAR dose

The planning dose of the bladder (V_45Gy,_ the percentage volume of received 45 Gy) was used as a reference when analyzing the relationship between bladder filling and the dose received by the bladder itself. The daily treatment dose of the bladder was initially normalized to the planning dose, which named RBV_45_(%), and then linearly fitted to the mean RVB to investigate the correlation between them.

The small bowel volume was defined as the volume in the specific range shown in Figure 1B. The small bowel volume at the time of treatment was called VS_treat_, whereas the small bowel volume at the time of planning was called VS_plan_. The RVB and the ratio of small bowel volume to planning small bowel volume (RVS) were statistically analyzed to investigate the effect of RVB on the small bowel volume.

$$\mathrm{RVS}=\left({\mathrm{VS}}_{\mathrm{treat}}/{\mathrm{VS}}_{\mathrm{plan}}\right)\times 100\%$$

### 2.7 Data analysis of the validation group

The validation set consisted of five patients with 25 total line CT images; the predicted values corresponding to RVB in all validation sets were calculated based on the simulation curves obtained from the study group. The absolute differences of predicted and actual values were analyzed.

### 2.8 Statistics

The SPSS 26.0 software was used for the t‐test of independent samples at a significance level of 0.05. Data were linearly fitted using Origin (Version 8.0, OriginLab, USA), and trend analyses were performed.

## 3 RESULTS

### 3.1 Changes in bladder volume during radiotherapy

**FIGURE 2:**
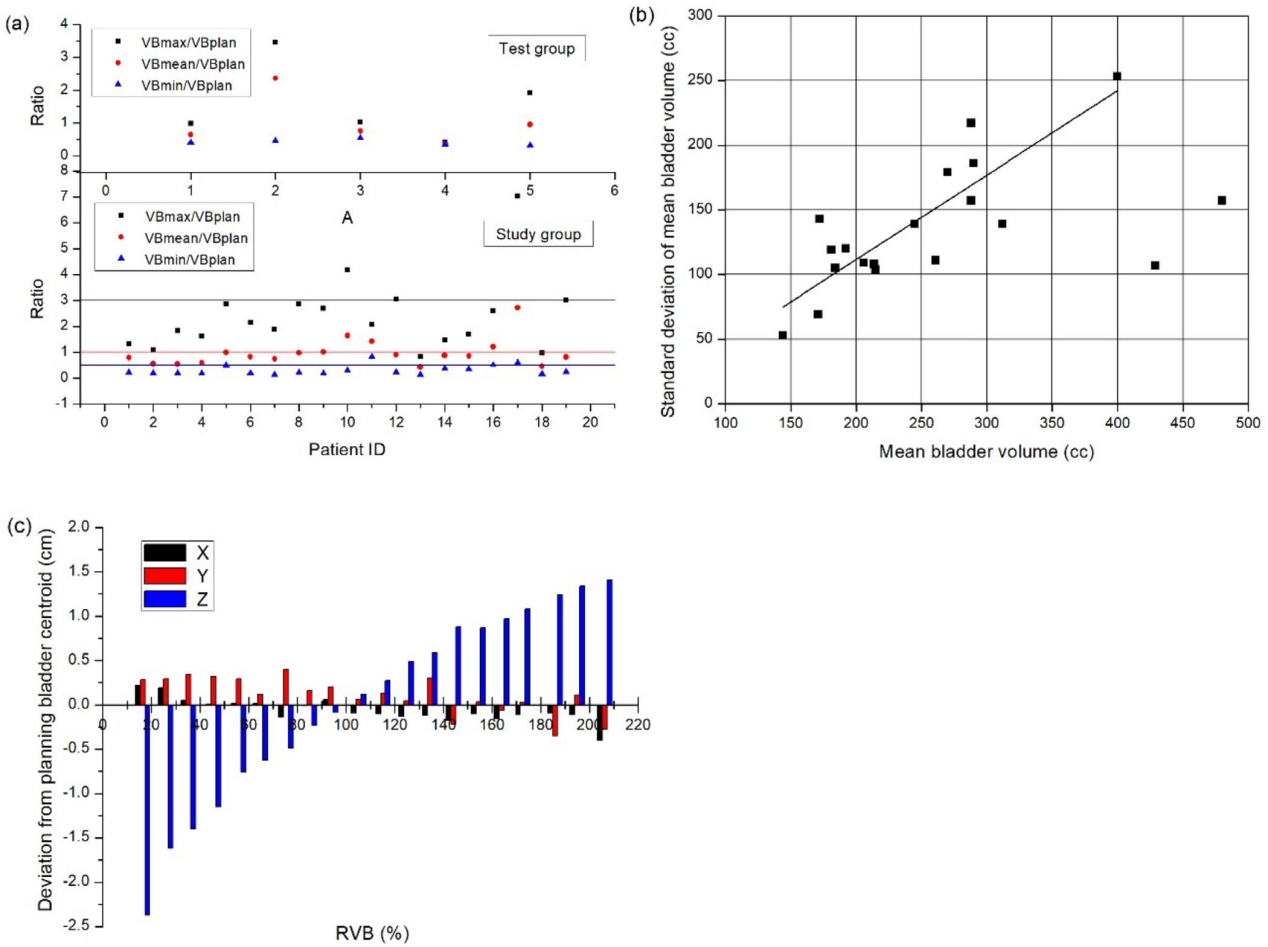
(a) Ratios of VB_max_, VB_min_, and VB_mean_ to VB_plan_, respectively, in 24 patients. (b) Bladder volume variability. Each point represents the effect of mean bladder volume on bladder volume variation in each patient as measured by standard deviation. The linear curve excludes the two points in the bottom right. (c) Bladder ISO deviation. X: left‐right; Y: anterior‐posterior; Z: superior‐inferior. RVB = (VB_treat_/VB_plan_) × 100%. VB_max_ = maximum bladder volume at treatment; VB_mean_ = mean bladder volume at treatment; VB_min_, = minimum bladder volume at treatment; RVB = ratio of bladder volume to planning bladder volume; VB_plan_ = planning bladder volume; VB_treat_ = bladder volume during radiotherapy.

Analyzed bladder volume changes in the study and validation groups showed that the mean bladder volume during treatment were smaller than the pretreatment in both the study group (259 ± 92 cm^3^ vs. 299 ± 107 cm^3^, *p* = 0.005) and validation group (315 ± 111 cm^3^ vs. 396 ± 151 cm^3^, *p* = 0.038; Table 1). In the study group, there was a large difference between the maximum bladder volume (VB_max_) and the minimum bladder volume (VB_min_) during treatment, among which 3 patients had a difference of about 15 times between the maximum and minimum volumes. The absolute volume difference was 720 cm^3^, 721 cm^3^ and 964 cm^3^ for the 3 patients. In addition, 15 patients (79.9%) had a VB_max_/VB_plan_ ratio of ≤3 (−64 to 486 cm^3^ for absolute volume difference), 16 patients (84.2%) had a VB_min_/VB_plan_ ratio of ≤0.5 (−387 to −107 cm^3^ for absolute volume difference), and 14 patients (73.7%) had a VB_mean_/VB_plan_ ratio of ≤1 (−232 to −1 cm^3^ for absolute volume difference) (Figure 2a). The standard deviation of the mean bladder volume per patient during treatment was positively correlated with the mean bladder volume (Figure 2b); the larger the mean bladder volume, the greater the deviation during treatment. The deviation of the bladder centroid from the planning centroid was further analyzed (Figure 2c). As the bladder volume increased, the bladder centroid shifted: −2.37 to +1.41 cm in the Z‐direction, −0.35 to +0.40 cm in the Y‐direction, and −0.40 to +0.22 cm in the X‐direction.

**TABLE 1:** Bladder volume information of the enrolled 24 patients.

	Study group (*N* = 19)		Validation group (*N* = 5)		
	Mean ± SD (cm^3^)	Min–Max (cm^3^)	Mean ± SD (cm^3^)	Min–Max (cm^3^)	*p*
Pretreatment	299 ± 107	109−542	396 ± 151	206−581	0.13
During treatment	259 ± 92	139−478	315 ± 111	194−464	0.19
*p*	0.005		0.038		

Abbreviation: SD, standard deviation.

### 3.2 Correlation of bladder filling with target volume dose coverage

**FIGURE 3:**
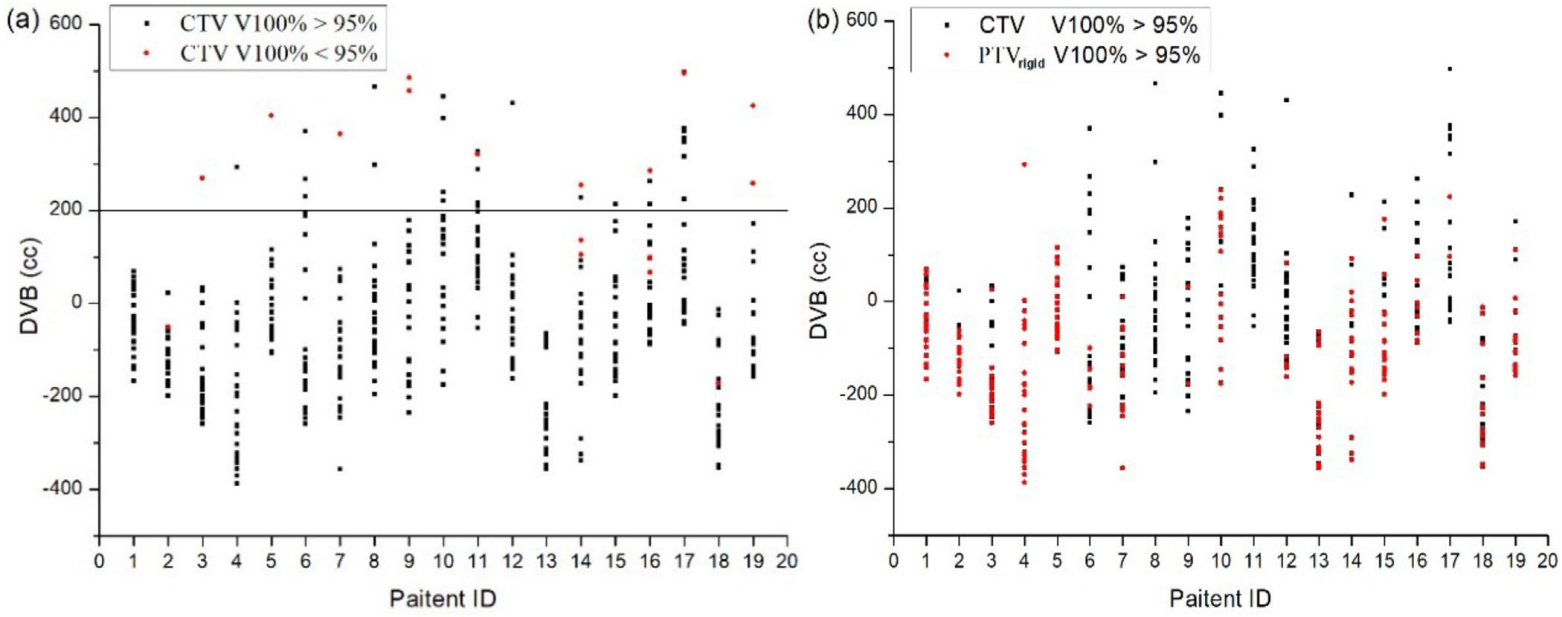
(a) CTV coverage for different deviations of bladder volumes. (b) CTV and PTV_rigid_ dose coverage for different deviations of the bladder volume. Red dots indicate V100% of <95% (a) and V100% of >95% (b) respectively. DVB = VB_treat_−VB_plan_. CTV = clinical target volume; PTV = planning target volume; DVB = deviation from the planning bladder volume; VB_plan_ = planning bladder volume; VB_treat_ = bladder volume during radiotherapy.

**FIGURE 4:**
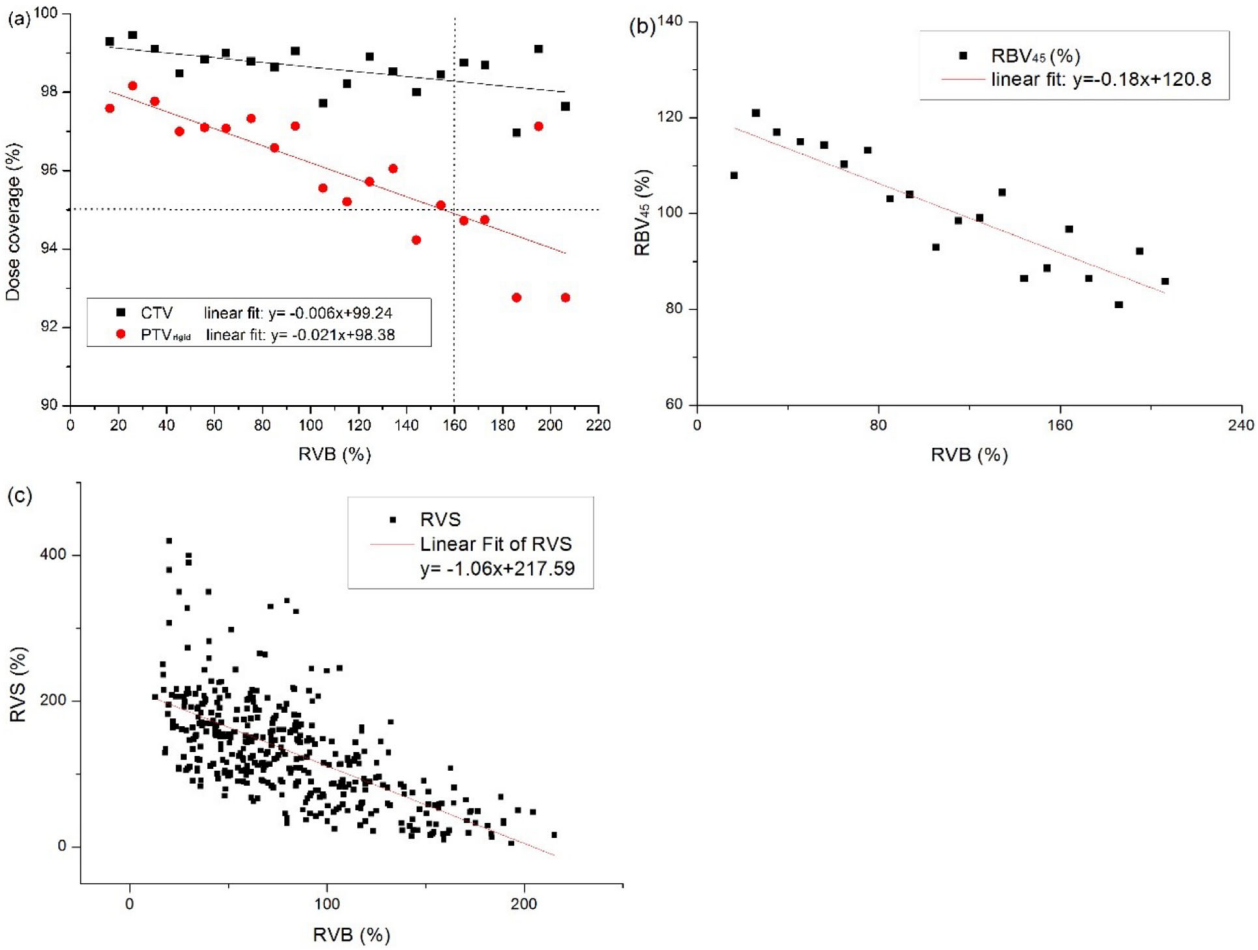
(a) Correlation of RVB with target dose. Each point represents the average of RVB in 10% step ranges. The two solid lines represent the linear fit curves for CTV, PTV_rigid_. (b) Correlation of RVB with bladder RBV_45_. (c) Correlation of RVB with small bowel RVS. RVB = (VBtreat/VBplan) × 100%, RVS = (VStreat/VSplan) × 100%. CTV = clinical target volume; PTV = planning target volume; RVB = ratio of bladder volume to planning bladder volume; RBV_45_ = ratio of treatment bladder dose to the planning bladder dose (V_45Gy_); RVS = ratio of small bowel volume to planning small bowel volume; VBplan = planning bladder volume; VBtreat = bladder volume during radiotherapy.

When the DVB was < 200 cm^3^ (92.2%, 438/475), 95% of the CTV was covered by the prescription dose in 98.6% (432/438) of the treatment sessions (Figure 3a). For PTV_rigid_, a large overlap with the points was observed with V100% delivered to > 95% of the CTV, representing a high conformity to CTV (Figure 3b). When the RVB was < 160%, a PTV_rigid_ coverage of > 95% met the clinical dose requirements. The prescribed dose coverages for CTV, PTV_rigid_ decreased with increasing RVB. The linear fitted equations for the RVB and prescribed dose coverage for each target volume were as follows (see also Figure 4a):

$$\begin{array}{ccc} & & \mathrm{CTV}:\;V100\%=-0.006*\mathrm{RVB}+99.24\\ & & \;\;\left(R^{2}=0.42,p=0.008\right)\\ & & {\mathrm{PTV}}_{\mathrm{rigid}}:V100\%=-0.021*\mathrm{RVB}+98.38\\ & & \;\;\left(R^{2}=0.65,p<0.001\right) \end{array}$$

### 3.3 Effect of bladder volume on bladder dose and small bowel volume

The bladder volume during treatment affects not only the dose coverage of the target volume but also the change in the dose to the bladder and the volume to the small bowel. The results showed that bladder RBV_45_ had an approximately negative linear correlation with RVB (Figure 4b), with a linear fitted equation of RBV_45_ = −0.18*RVB + 120.8 (R^2^ = 0.80, *p* < 0.001). The specific range of small bowel volume also had a negative linear correlation with RVB (Figure 4c). In the region of interest, the small bowel volume decreased as the bladder volume increased, with a linear fitted equation of RVS = −1.06*RVB + 217.59 (R^2^ = 0.41, *p* < 0.001). A full bladder pushes the small bowel out of the pelvis, which in turn reduces the irradiated volume of the small bowel.

### 3.4 Validation group

Based on the linear fitted equation obtained in the study group, the predicted dose and actual dose on daily‐CT images of 5 patients in the validation group were compared to validate the change in target volume coverage, bladder dose, and small bowel volume.

The verification results showed that 1) the linear prediction model could predict the effect of bladder volume change on target volume coverage, and the deviation between the predicted values and the true values of all parameters was small. The predicted and true values of CTV and PTV_rigid_ prescription dose coverage were (98.76% ± 0.27% vs. 97.94% ± 1.18%, *p* = 0.62) and (96.69% ± 0.96% vs.95.18% ± 2.51%, *p* = 0.54), respectively. The maximum deviations were 2.70% and 5.97%, respectively. In addition, the mean deviation of prescription dose coverage for the two target volume in all validation sets was 1.21% to 2.46%. 2) The linear prediction model can also better predict the effect of bladder volume change on the RBV_45_ of bladder and RVS of small intestine. The predicted and true values of bladder RBV_45_ and RVS were (106.33% ± 8.22% vs. 108.78% ± 17.69%, *p* = 0.44) and (132.36% ± 36.80% vs.108.53% ± 38.54%, *p* = 0.13), respectively. The mean deviations were 12.12% and 31.67%, respectively. (See Table 2).

**TABLE 2:** Absolute errors of test results with predicted values.

Parameter prediction	Predicted value	Actual value	Absolute error (%)			*p*
	Mean ± SD	Mean ± SD	Mean	Min	Max	
CTV (%)	98.76 ± 0.27	97.94 ± 1.18	1.21	0.05	2.70	0.62
PTV_rigid_ (%)	96.69 ± 0.96	95.18 ± 2.51	2.46	0.40	5.97	0.54
RBV_45_ (%)	106.33 ± 8.22	108.78 ± 17.69	12.12	1.27	27.17	0.44
RVS (%)	132.36 ± 36.80	108.53 ± 38.54	31.67	9.48	53.85	0.13

Abbreviations: CTV, clinical target volume; PTV, planning target volume; RBV_45_, ratio of treatment bladder dose to the planning bladder dose (V_45Gy_); RVS, ratio of small bowel volume to planning small bowel volume; SD, standard deviation.

## 4 DISCUSSION

There are many sources of uncertainty in radiotherapy for cervical cancer, among which bladder filling is one of the significant ones. Deviations in bladder volume can significantly impact the target volume dose coverage and the relationship between volume changes in the bladder and small bowel.^12^ Different radiotherapy centers have different guidelines for bladder filling in patients with cervical cancer .^9^, ^15^ In addition, the lack of reproducibility of patient bladder filling prior to each treatment adds to the complexity of target volume and OAR dosimetry. Several studies^8^, ^16^, ^17^ have analyzed the effects of bladder filling on changes in pelvic organ positions using various imaging techniques such as CBCT, MRI, and ultrasound, etc., but there are no studies based on daily online CT. Recently, a retrospective study has reported the use of CBCT to examine dose changes in the target volume during adaptive radiotherapy in patients with cervical cancer.^18^ V95% of PTV and CTV from the online adaptive replan (ART) was changed on an average of 9.2% (SD = 14.3) and 7.9% (SD = 13.8) compared to the original plan recalculated on CBCT anatomy (IGRT). The D2cc to the bladder and bowel for each fraction changed an average of −0.02 Gy (SD = 0.09) and −0.08 Gy (SD = 0.06), respectively. But they did not analyze the factors affecting the dose distribution. Our study analyzed the dose deviations between the original plan recalculated on daily CT and the original plan calculated on simulation CT. The results showed that the prescribed dose coverage in the CTV was > 95% when DVB was < 200 cm^3^, whereas that in the PTV was > 95% when RVB was < 160%. Another factor affecting the target location and dosimetry of radiotherapy for cervical cancer is rectal filling status. Several studies reported the anterior‐posterior and superior‐inferior movements of cervix‐uterine motion due to rectal filling.^19^, ^20^, ^21^ A greater impact of rectal filling on cervical and upper vaginal motion compared with the uterus was noted.^20^ There was an inverse relationship between rectal anterior‐posterior diameter and bladder volume.^9^ Thus, the impact of rectal filling on cervix‐uterine motion may be insignificant compared to the impact of increasing bladder volume on cervix‐uterine motion. Furthermore, the impact of rectal filling was minimized after rectal preparation at each treatment.

We used the daily‐CT images (FBCT) obtained using the uRT‐linac506c, or the first time to our knowledge, the effect of bladder volume change on target volume dose coverage in patients with cervical cancer, analyzed the pattern of deviation of the bladder with different filling grades, as well as the relationship between volume changes of the bladder and small bowel, and established linear fitted equations to predict the target volume coverage, irradiated dose and volume of OARs based on the bladder volume change. In our study, the small number cases (5.9%, 28/475) when RVB ≥ 210% have large bladder volumes which may not be reproducible in the treatment course. We aimed at finding general dose prediction models related to bladder volumes. Thus, the extreme cases in which VB_treat_ were considerably larger than VB_plan_ (RVB ≥ 210%) were not considered.

Regarding the pattern of deviation in bladder volume, although the patients were instructed to hold urine prior to treatment, the reproducibility of the bladder volume remained a problem due to various factors, such as inconsistent patient wait time, inconsistent water consumption prior to treatment, and worsening of bladder function with increasing radiation dose (Table 1, Figure 2a). As shown in Figure 2b, the larger the mean bladder volume, the more difficult it is to maintain the same bladder volume during treatment, which suggests that bladder overfilling should be avoided to improve reproducibility during positioning and treatment, which is consistent with the findings of Chen et al.^15^ Different degrees of bladder filling lead to changes in its position, with a complex filling pattern, not just a fixed centroid expanding in all directions but linked to the bladder volume itself. Figure 2c shows the displacement of the bladder centroid in three directions according to the bladder volume ratio, with the most pronounced displacement of the centroid observed in the superior‐inferior direction and a relatively insignificant displacement in the other two directions as the bladder volume changes during treatment. Compared with the planning bladder centroid, the daily bladder centroid shifted toward the superior side when the bladder volume increased during treatment, with the displacement ranging from −2.37 to +1.41 cm.

Regarding the effect of the bladder on the target volume dose, when the DVB was < 200 cm^3^ (92.2%, 438/475), the prescribed dose coverage of the CTV was generally maintained above 95% (98.6%, 432/438); with increasing differences, the target volume coverage becomes considerably worse (Figure 3a). When the bladder volume is extremely large, the uterus and cervix are affected and eventually displaced.^22^, ^23^, ^24^, ^25^ The slopes of the two fitted curves also showed that the doses of CTV and PTV_rigid_ decreased to various degrees with increasing bladder volume during treatment; the bladder volume affected the doses of the PTV_rigid_ to a substantially higher extent compared with that of the CTV (Figure 4a). When the RVB was < 160% and the PTV_rigid_ coverage was > 95%, the planning CTV‐PTV (PTV_rigid_) expansion distance was sufficient to better enclose the deformed CTV.

A previous study of 166 patients with locally progressive cervical cancer showed that advanced radiation cystitis was less likely to occur and milder when the planning bladder volume ranged from 100 to 150 cm.^3^, ^26^ The relationship between bladder volume and dose during radiotherapy must be elucidated further to reduce the risk of subsequent complications. This study was the first to find a linear relationship (RBV_45_ = −0.18*RVB + 120.8) between bladder RBV_45_ and RVB by analyzing 475 online CT plans. Validation of this linear model showed that the actual values after normalization were in good agreement with the predicted values, so this model may be used as a clinical reference for predicting the effect of bladder volume change. In addition, the small bowel dose in cervical cancer radiotherapy remains controversial. The influence of small bowel physiological peristalsis and bladder volume between fractionated treatments can lead to changes in the position of the small bowel during treatment and adds uncertainty to the actual small bowel dose. We performed a linear fit of RVS to RVB for all patients to study the effect of bladder volume deviation on small bowel volume changes and this study can provide a model for the effect of bladder volume change on small intestine volume and dose exposure.

This study has three major limitations. First, due to the limitation that our group does not have access to the commercially available dose deformation and accumulation tools, we only analyzed the dose deviation of single radiotherapy without deformation and accumulation the actual total dose of exposure. Therefore, we could not support the generalization of the conclusion for utilization in adaptive radiotherapy for cervical cancer patients. In the future, further research on this issue will be conducted. Second, this study only analyzed the correlation of bladder filling with dose distribution but did not study the impact of change in dose distribution on clinical prognosis (such as tumor control probability and normal tissue complication probability). Third, due to the limited number of patients, we just established a basic correlation model of bladder volume and dose distribution. Furthermore, we will increase the sample size to establish a more reliable model.

## 5 CONCLUSION

Our study analyzed the effects of bladder volume changes on target and OAR volumes and dosimetry during cervical cancer radiotherapy based on daily online CT images first to our knowledge. We established a quantitative relationship between these parameters, thereby providing linear models to predict parameters for radiotherapy of patients with cervical cancer.

## AUTHOR CONTRIBUTIONS

Manuscript: F.Z., M.Z., G.W., Y.L.P., Y.M.L.; Sample acquisition and patient cohort: K.C., Z.Y.Q., X.W.D., X.T.L.; Statistical analysis: L.Y., L.H.D., K.C. All authors read and approved the final manuscript.

## CONFLICT OF INTEREST STATEMENT

The authors declare no potential conflicts of interest.

## Data Availability

The datasets are backed up on the Research Data Deposit (RDD Number: RDDA2022141152, https://www.researchdata.org.cn) and are available upon reasonable request.
